# Assessment of Subjective Tinnitus Treatment Results Using a Prototype Device for Electrical and Magnetic Stimulation of the Ear-Preliminary Study

**DOI:** 10.3390/life12060918

**Published:** 2022-06-19

**Authors:** Jurek Olszewski, Marzena Bielińska, Andrzej Julian Kowalski

**Affiliations:** Department of Otolaryngology, Laryngological Oncology, Audiology and Phoniatrics, II Chair of Otolaryngology, Medical University of Lodz, 90-549 Lodz, Poland; marzena.bielinska@umed.lodz.pl (M.B.); andrzej.kowalski.1@umed.lodz.pl (A.J.K.)

**Keywords:** treatment, subjective tinnitus, prototype device, electrical and magnetic stimulation of the ear

## Abstract

**Background**: The aim of the study was to evaluate the effectiveness of subjective tinnitus treatment in patients with cochlear sensorineural hearing loss with magnetic ear stimulation using a prototype device. Since the 1970s, studies have been conducted on the use of electrical stimulation of the ear in the treatment of tinnitus. The available literature contains various hypotheses about the influence of electrical stimulation of the ear on tinnitus. **Material and Methods**: Preclinical studies were performed for 100 patients, 40 women and 60 men (124 ears in total), aged 38–72 years, treated for tinnitus. A subjective assessment of the loudness of tinnitus was performed, and the frequency and intensity as well as hearing threshold were determined using a prototype device for electro-magnetic stimulation of the ear. The treatment cycle consisted of 10 five-minute stimulations performed daily 5 times a week. **Results:** Before treatment, persistent tinnitus was found in 100 ears (80.6%) and periodic tinnitus in 24 ears (19.4%). Immediately after treatment, persistent tinnitus was present only in 50 ears (40.3%) and periodic tinnitus in 40 ears (32.3%). Complete resolution of tinnitus was noted in 34 ears (27.4%). On the other hand, the examination performed 3 months after the treatment showed persistent tinnitus in 40 ears (32.3%) and periodic tinnitus in 50 ears (40.3%), and complete resolution of tinnitus was recorded in 34 ears (27.4%). Based on the VAS analog scale, there was an improvement in tinnitus in 98 ears (79.0%) immediately after treatment and no improvement in 26 ears (20.0%). The mean VAS scale before treatment was 4.9 points, after treatment it was 2.1 points and 3 months after treatment it was 1.9 points. **Conclusions:** The preliminary research results show the high effectiveness of magnetic stimulation in the treatment of tinnitus with the use of a prototype device for electromagnetic stimulation of the ear. There was no negative effect of the stimulation on hearing or tinnitus.

## 1. Introduction

Since the 1970s, studies have been conducted on the use of electrical stimulation of the ear in the treatment of tinnitus. The available literature contains various hypotheses about the influence of electrical stimulation of the ear on tinnitus: Latkowski et al. [1] point to the increase in the flow of neurotransmitters in synapses, while Portmann et al. [2] indicate a modification of the electric potential of the cochlea, whereas Watanabe et al. [3] emphasize the improvement of blood flow in the inner ear or the synchronization of discharges in the fibers of the auditory nerve. However, the most comprehensive model for electrical stimulation of the cochlea and the upper levels of the auditory pathway and their impact on tinnitus is the model of electrical stimulation with the use of auditory implants (mainly cochlear implants).

Owing to the animal model of tinnitus (cochlear damage induced by noise or ototoxic drugs), the influence of electrical stimulation on the cells of the cochlea and other levels of the auditory pathway was investigated, demonstrating a beneficial effect in relation to these structures and behavioral signs of tinnitus in animals [4,5,6,7].

Numerous world literature data indicate the effectiveness and safety of the use of electrical and magnetic stimuli in the treatment of subjective tinnitus. Electrical stimulations are (or were) performed along the entire auditory pathway, i.e., from stimulation of the external ear to direct intracranial stimulation of the auditory cortex, applying both invasive and non-invasive procedures [8]. Direct invasive transtympanic cochlear electrostimulation procedures were performed through the round window (through the promontory) or with the use of a cochlear implant [2,3,9,10]. Aran et al. [9] observed differences in results depending on the type of stimulation, and, in the case of placing the electrode in the niche of the round window, they obtained a 60% improvement and only 43% on the promontory. Then, electrostimulation is used in the promontorium, within the round window, or by immersing the electrode in the external ear canal in a saline solution [11]. A similar effect was observed by Portmann et al. [2]. In their research, in each case, stimulation which involved placing the electrode in the niche of the round window gave a better therapeutic effect. An additional application of electrical stimulation of the hearing organ is its use for diagnostic purposes, prior to implantation of cochlear implants. Then, electrostimulation is used in the promontorium, within the round window, or by immersing the electrode in the external ear canal in a saline solution.

In 1973, for the first time at the House Ear Institute, tinnitus relief was reported following implantation of a single-electrode cochlear implant. Quaranta et al. [12] reviewed publications on this subject and estimated the incidence of tinnitus in subjects qualified for cochlear implantation due to profound hearing loss or deafness at 66–86%. Depending on the authors, the effectiveness in reducing or resolving tinnitus nuisance varies from 67–79% [12,13].

Transcutaneous electrodes have been used by Steenerson et al. [14] and Zeng et al. [10], the idea being to treat tinnitus non-invasively. In a group of 20 patients, Engelberg et al. [15] observed improvement in 82% of cases.

Using different electrode locations, the influence of the type of current applied on the result of electrostimulation has been investigated. Aran et al. [9] used an alternating current with positive polarity. Portman et al. [2] compared the effects of currents with positive and negative polarities.

There are considerable datasets available in the international literature on the effectiveness of this method, using direct and alternating current, without any damaging effects of this therapy on the central nervous system and the auditory pathway having been found [16,17,18].

Transcranial magnetic stimulation (TMS) has been used in medicine for several decades. It is a non-invasive method affecting the excitability of the cerebral cortex, owing to which it has gained increasing attention as a therapeutic tool in a wide spectrum of neuropsychiatric disorders [19,20,21,22,23]. Low-frequency repetitive transcranial magnetic stimulation (rTMS), e.g., 1 Hz, effectively reduces cortical activity, especially in the areas of increased excitability. Numerous clinical studies have shown the efficacy of transcranial magnetic stimulation of the temporal lobe as a therapeutic method in relation to tinnitus [24,25,26]. Typically, TMS is applied using a figure-8 coil over the temporal region. This method directly affects the activity of the superficial cortex and indirectly affects areas functionally connected with stimulated areas, e.g., the thalamus [27]. With positron emission tomography, double-cone coil TMS has been shown to modulate the activity of deeper brain structures as well as those located more peripherally to the stimulated areas [28].

The biological effect of TMS depends on the stimulating frequency of the magnetic field as well as on the moment at which the results of stimulation and magnetic coil orientation are assessed. The analysis of motor cortex stimulation showed that low-frequency magnetic fields (<1 Hz) have inhibitory properties, whereas high-frequency fields (<3 Hz) have stimulating effects on neurons. Moreover, the immediate effect of the stimulation is believed to depend on the direct reaction of neurons to the stimulus, while the later, indirect one (observed after minutes) depends on the biochemical changes occurring in the stimulated area. Therefore, the use of low-frequency transcranial magnetic stimulation is justified in the treatment of tinnitus due to its inhibitory potential in the stimulated cerebral cortex and functionally related regions of pathological, increased activity (e.g., as a result of cochlear hearing damage), which is most likely the cause of tinnitus. On the other hand, with regard to the peripheral part of the hearing organ, the use of frequencies in the frequency range represented in the hearing organ, i.e., 20 Hz–20 kHz, is justified.

Electrical and magnetic stimulation of the ear is a method that targets the cause of tinnitus, most frequently found in the inner ear (in the cochlea). An innovative prototype device for electrical and magnetic stimulation of the ear has been specially designed and constructed for the diagnosis and treatment of subjective tinnitus at the Department of Otolaryngology, Laryngological Oncology, Audiology and Phoniatrics of the Medical University in Łódź. Thanks to the computer program cooperating with the device, it was possible to individually design a therapeutic stimulus (in terms of electrical or magnetic impulse) adapted to tinnitus. Owing to the non-invasive procedure, stimulation can be repeated as a treatment cycle, thanks to which a stimulus accumulation effect is obtained, which leads to neuroplastic changes and neuromodulation.

The design of the device was based on the many years of experience of the authors in the use of ear stimulation in the treatment of tinnitus [29,30,31,32]. Thanks to this experience, the device responds to the highly individualized needs of patients, making it possible to adjust the therapeutic stimulus. Thus, owing to ear stimulation, we can influence the cause of tinnitus, and our previous studies have shown that by giving a stimulus in this way we can also modulate the bioelectrical activity of the brain, which in patients with tinnitus undergoes secondary abnormal changes.

The innovative prototype device responds to the huge clinical demand, and thanks to the obtained European patent (granted by the European Patent Office on 27 January 2021, no. 3498166, for an innovative prototype device for electromagnetic ear stimulation in the treatment of subjective tinnitus), it also has great commercial potential. The patent was validated in the following European countries: Germany, France, Great Britain, Italy and Poland.

This device—as mentioned above—allows the simultaneous use of electro- and magneto-stimulation of the ear. The creation of a magnetic coil to stimulate the cochlea is a completely innovative solution. Thanks to a dedicated computer program with which the pacemaker cooperates, completely individualized treatment can be programmed by selecting the characteristics of stimuli according to the needs of patients. What is more, an innovative system to stabilize the electrode or the stimulating coil in the external auditory canal (EAC) can perform stimulation without having to hold the parts in the patient’s ear. Until now, there has been no device dedicated to this type of therapy in individuals with subjective tinnitus available on the Polish or international market.

The aim of the study was to evaluate the effectiveness of the treatment of subjective tinnitus in patients with cochlear sensorineural hearing loss using the method of magnetic ear stimulation with the use of a prototype device.

## 2. Material and Methods

Preclinical studies were conducted in 100 patients, including 40 women and 60 men, aged 38–72 years (mean: 58.5 years), receiving treatment for tinnitus at the Department of Otolaryngology, Laryngological Oncology, Audiology and Phoniatrics of the Military Medical Academy Memorial Teaching Hospital in Łódź. A total of 124 ears were examined, of which 24 had bilateral and 100 had unilateral tinnitus, 60 ears on the left side and 40 ears on the right side.

The methods included: detailed medical history, otoscopy, and comprehensive audiological diagnostics, which included: recruitment test SISI (Short Increment Sensitivity Index), verbal audiometry, impedance audiometry, auditory brainstem response (ABR) and measurements of otoacoustic emission (TEOAE, DPOAE and SOAE). Audiological tests were supplemented with electronystagmography (ENG) or videonystagmography (VNG) and diagnostic imaging. A subjective assessment of tinnitus loudness was also performed; the frequency and intensity as well as the hearing threshold were determined.

**Figure 1:**
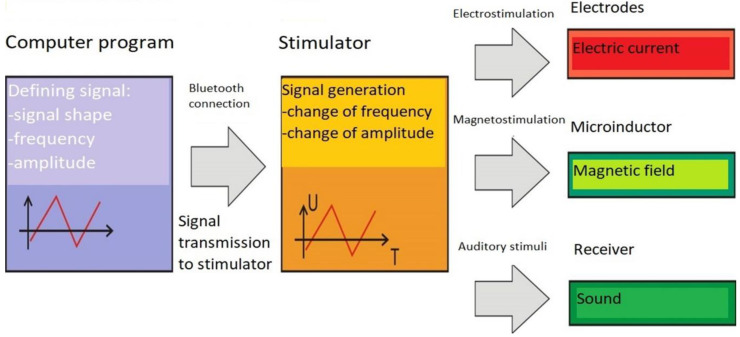
Construction and working principle of the prototype device for electromagnetic ear stimulation.

The treatment cycle consisted of 10 five-minute stimulations performed daily 5 times a week. A prototype device for electromagnetic stimulation of the ear was used for the treatment (Figure 1).

The prototype device for electrical and magnetic stimulation of the ear for clinical use consists of the following elements: a computer program, a stimulator, a handset equipped with a stimulation electrode and a microinductor of the magnetic field. It has been assumed that the program for defining signal parameters will be installed on a computer equipped with a modem working with standard Bluetooth, thanks to which it will be possible to send signal parameters wirelessly to the stimulator.

Furthermore, this device underwent safety tests, consisting of two elements, electrical safety and electromagnetic compatibility, in accredited laboratories of the Research, Attestation and Certification Center in Gliwice, within the concluded contract, no. OBAC/2545/JZO, of 27 March 2020.

The use of low-frequency transcranial magnetic stimulation is justified in the treatment of tinnitus due to its inhibitory potential in relation to the areas of the stimulated cerebral cortex and areas functionally related thereto with pathological increased activity. On the other hand, with regard to the peripheral part of the hearing organ, the justification will be for frequencies in the frequency range represented in the hearing organ, i.e., 20 Hz–20 kHz.

The stimulating coil was placed in the external auditory canal (EAC). For safety reasons, a stimulus with a frequency of 20 Hz was applied. The stimulation range is 20 Hz–20,000 Hz, magnetic induction is in the range of 0–28 mT and the intensity of the current is 0–2 mA. These are parameters (ranges of values) considered safe to use in the head region. Individually, the frequency of the stimulus will be adjusted to the frequency of the patient’s tinnitus and the intensity to the patient’s individual tolerance; the maximum value will be 2 mA. The stimulation time is 5 min for the ear.

Tinnitus was assessed before treatment, immediately after the end of treatment and after 3 months using the visual analogue scale (VAS) for loudness and pure-tone threshold audiometry.

Statistica (version 8.1) and Student’s *t*-tests were used for statistical analysis. *P*-values < 0.05 were considered statistically significant.

The study was approved by the Bioethics Committee (RNN/278/20/KE). All patients gave their informed consent to participate in the project.

## 3. Results

**Figure 2:**
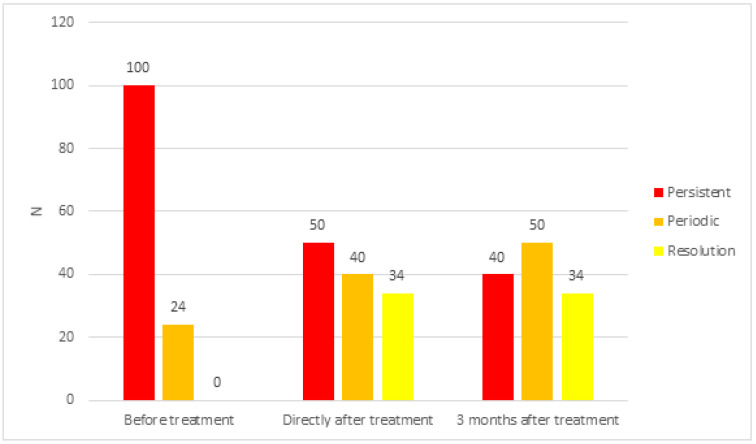
Characteristics of tinnitus in the study group before and after treatment.

In the investigated ears (Figure 2) before treatment, persistent tinnitus was found in 100 ears (80.6%)—31 female (25.0%) and 69 male ears (55.6%)—and periodical tinnitus in 24 ears (19.4%)—7 female (5.7%) and 17 male ears (13.7%).

Immediately after treatment, persistent tinnitus was present in only 50 ears (40.3%)—20 female (16.1%) and 30 male ears (24.2%)—and periodical tinnitus in 40 ears (32.3%)—15 (12.1%) female and 25 male ears (20.2%). Tinnitus completely subsided in 34 ears (27.4%)—14 female (11.3%) and 20 male ears (16.1%).

Three months after the treatment, persistent tinnitus persisted in 40 ears (32.3%)—15 female (12.1%) and 25 male ears (20.2%)—and periodical tinnitus in 50 ears (40.3%)—20 female (16.1%) and 30 male ears (24.2%). Complete resolution of tinnitus was noted in 34 ears (27.4%)—14 female (11.3) and 20 male ears (16.1%).

Immediately after the end of the treatment, improvement was achieved in 78 ears (62.9%)—28 female (22.6%) and 50 male ears (40.3%)—in the form of a reduction of tinnitus loudness by 50–85%. Tinnitus did not change in 22 ears (17.7%)—8 female (6.4%) and 14 male ears (11.3%). No intensification of symptoms was observed in any of the subjects.

**Figure 3:**
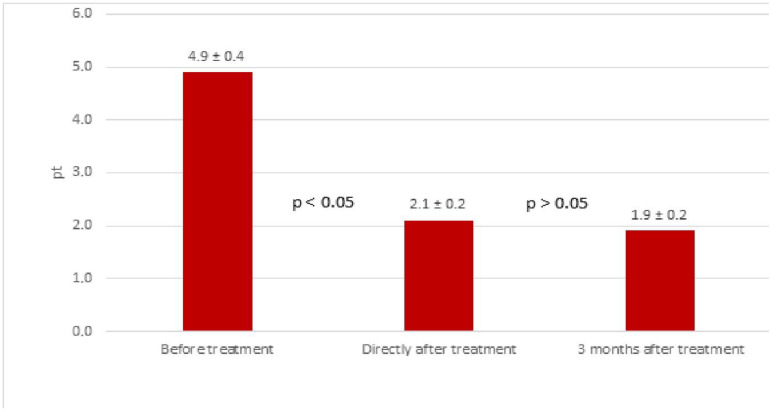
Results of tinnitus treatment (loudness) in the study group based on the VAS scale before and after treatment (mean values in points).

Based on the VAS scale, tinnitus improved in 98 ears (79.0%) immediately after treatment—34 female (27.4%) and 64 male ears (51.6%). No improvement was found in 26 ears (20.0%)—10 female (8.1%) and 16 male ears (12.9%). The mean VAS before treatment was 4.9 ± 0.4 points, immediately after treatment it was 2.1 ± 0.2 points and 3 months after treatment it was 1.9 ± 0.2 points (Figure 3).

**Figure 4:**
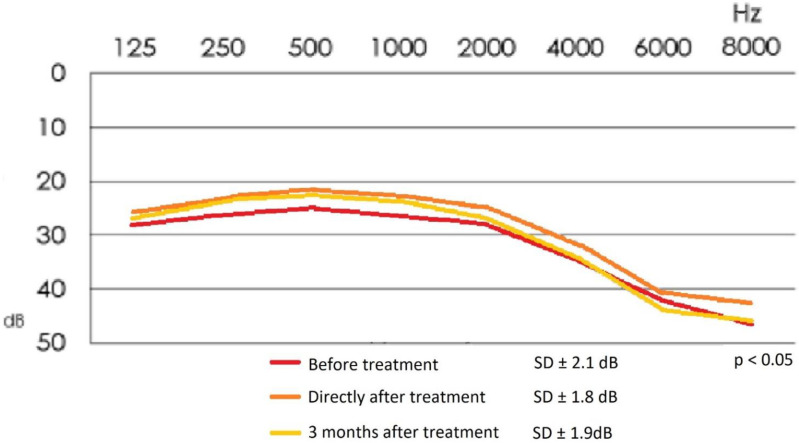
Mean audiometric hearing threshold values in the study group before and after treatment.

Figure 4 demonstrates mean values of the audiometric hearing threshold in the studied group of patients before and after the treatment.

The mean values of the audiometric hearing loss for the frequencies of 500 Hz, 1000 Hz and 2000 Hz are as follows: before treatment: 27.6 ± 2.1 dB; directly after treatment: 23.6 ± 1.8 dB; 3 months after treatment: 24.3 ± 1.9 dB.

## 4. Discussion

Despite numerous studies and clinical research, no 100% effective method of treatment for tinnitus has been established. Various methods of treatment are offered, including pharmacotherapy, electrostimulation, hyperbaric oxygen chambers, laser therapy, tinnitus sound masking with the use of tinnitus masking devices, hypnosis, etc. [29,30,31,32,33]. Among the methods of treatment, the most frequently mentioned is Tinnitus Retraining Therapy (TRT), which has been used for 25 years. TRT offers significant improvement for about 80% of patients.

In the authors’ own research [33], among elderly patients suffering from tinnitus and concomitant sensorineural hearing loss resulting from senile deafness, hearing prostheses have been used and, above all, causal treatment of existing organic changes. Pharmacological treatment with vascular drugs, a low-cholesterol diet, fighting overweight, increased physical activity and avoiding noise and silence were recommended. Attempts at treatment requiring active auditory training in this group of patients encountered difficulties and incomprehension, as did the recommendation to purchase a noise generator, which was probably related to the costs incurred when purchasing a device not fully reimbursed by the National Health Fund. Systemic cryotherapy [33] in combination with intensive kinesiotherapy is used in all patients with radiographic degenerative changes in the cervical spine.

In patients with dizziness and tinnitus, pharmacological treatment and kinesiotherapy are recommended as supportive therapies.

Øreblue^®^ is a new treatment method for tinnitus and hyperacusis combining auditive rehabilitation and psychological therapy [34]. Despite the use of various alternative methods of tinnitus treatment in many centers around the world, therapy is still not very effective and constitutes a serious clinical problem [35,36,37].

Thanks to the research conducted with the use of a prototype device for the therapy and diagnosis of tinnitus, it will be possible to approximate the mechanism of its generation. However, more importantly, the preliminary results of the research indicate that by introducing a prototype stimulator into clinical use it is possible to achieve a significant improvement in the effectiveness of the treatment of this symptom. This, in turn, should reduce the number of medical consultations (ENT, audiological, neurological, internal medicine and psychiatric) and costs related to treatment. Modifications of this device to a form in which the equipment can be used by the patient at home will support the above-mentioned activities as well as significantly reduce the queues of patients waiting for diagnosis and treatment, especially in highly specialized teaching hospitals.

In order to use the device at home, the patient needs to undergo a week-long training in its use in our department. Each patient also receives contact details for technical support and for the doctor supervising the examination, to whom the patient can refer in case of any problems.

The TRT (Tinnitus Retraining Therapy) method is based on a neurophysiological model of tinnitus generation. It is characterized by a very high efficiency unattainable in the therapies used so far. Research shows that TRT therapy is effective in as much as 85% of cases.

At the current stage of preliminary research, our method using a prototype device cannot be compared with TRT.

The possibility of a patient carrying out the therapy by his or herself at home will additionally have a positive effect on the treatment, e.g., the patient will avoid tiring commutes to hospital—and in the case of our department, patients come from all over Poland—and will not have to bear the costs of these journeys, nor will they have to take sick leave to receive treatment for their tinnitus.

The prototype device will be used by the patient in the future, after registration with the Registration Office of Medicinal Products.

Nationally, taking into account the prevalence of tinnitus (about 15% of the adult population as well as children), these measures can improve the quality of life of people suffering from tinnitus and reduce the financial costs to this group of patients.

The prototype device for electrical and magnetic stimulation of the ear has been classified as a class IIa medical device in accordance with classification rule 9 of the Regulation (EU) of the European Parliament and of the Council 2017/745 of 5 April 2017.

After obtaining the consent of the Office for Registration of Medicinal Products of the Republic of Poland, a randomized, double-blind, placebo-controlled clinical trial has been planned for a large population of 300 patients with subjective tinnitus in the period 01.12.2021 to 28.02.2026. Electrostimulation (ES) and magnetic stimulation (MS) of the ear will be carried out by a non-invasive method by inserting an electrode (E) or coil (C) into the external auditory canal (EAC) using a prototype device for electromagnetic ear stimulation. In the group receiving combined electrical and magnetic stimulation, MS will be performed first, then ES, with 5 min between stimulations. In the placebo group, the procedure will be analogous: the electro-magneto-stimulation device will be turned on but the stimulation will not be activated. Stimulation time (ES/MS) will be for 5 min and unilateral or bilateral depending on whether the tinnitus is unilateral or bilateral. Procedures will be performed daily, on working days, 10 times in total. Stimulation will be in the human audible frequency range of 20 Hz–20 kHz, the alternating current used will have an intensity of 0–2 mA and the magnetic field will have an induction of 0–28 mT. The study is still ongoing.

To assess the effect of tinnitus treatment in the planned clinical trial, apart from the VAS scale, other questionnaires will be used, such as TQ, THI or TFI.

## 5. Conclusions

As already mentioned, further research on the effectiveness of the prototype device for electrostimulation of the ear in patients with tinnitus is necessary. The results of the initial studies are encouraging. Preliminary research results show the high effectiveness of magnetic stimulation in the treatment of tinnitus with the use of a prototype device for electromagnetic stimulation of the ear. In addition to evaluating the effectiveness of therapy with this device, it is also very important to assess the safety of its use. The safety tests that have been carried out have shown that there was no negative effect of the stimulation on hearing or tinnitus, and no serious adverse events were observed.

## Data Availability

Data are available upon request from the corresponding author.
